# Advancing Surgical Management of Penile Cancer: Single Port Bilateral Inguinal Lymph Node Dissection

**DOI:** 10.1590/S1677-5538.IBJU.2024.0663

**Published:** 2025-01-22

**Authors:** Sisto Perdonà, Alessandro Izzo, Antonio Tufano, Francesco Passaro, Giuseppe Quarto, Achille Aveta, Roberto Contieri, Savio Domenico Pandolfo, Riccardo Autorino, Gianluca Spena

**Affiliations:** 1 Istituto Nazionale Tumori di Napoli IRCCS Fondazione "G. Pascale" Department of Urology Napoli Italy Department of Urology, Istituto Nazionale Tumori di Napoli IRCCS Fondazione "G. Pascale", Napoli, Italy; 2 University of L’Aquila Department of Life, Health and Enviromental Sciences L’Aquila Italy Department of Life, Health and Enviromental Sciences, University of L’Aquila, L’Aquila, Italy; 3 Rush University Medical Center Department of Urology Chicago IL USA Department of Urology, Rush University Medical Center, Chicago, IL, USA

## Abstract

**Introduction:**

Penile cancer is a rare but aggressive malignancy, with inguinal lymph node involvement representing a key prognostic indicator (1, 2). NCCN guidelines recommend prophylactic inguinal lymph node dissection (ILND) for intermediate-to-high-risk patients (pT1b, ≥T2) with non-palpable nodes, aiming for early staging and improved outcomes (3).

The SP-approach employs a single incision and advanced robotic instrumentation to enhance maneuverability, reduce morbidity, and optimize recovery.

Widely used in kidney and prostate surgery (4, 5), this is, to our knowledge, its first application for ILND in Europe.

**Material and Methods:**

This video shows a novel robotic-assisted bilateral, superficial and deep ILND using the DaVinci SP™ system.

In this patient, a preoperative 3D reconstruction allowed detailed visualization of lymph nodes and surrounding structures, enabling precise dissection and an improved intraoperative orientation using Tilepro feature.

**Results:**

Compared to open techniques, robotic ILND offers similar lymph node yields with superior cosmetic outcomes and reduced postoperative pain (6). These benefits are amplified with the SP system, which excels in the constrained inguinal region by minimizing instrument interference and enhancing efficiency (7). Fewer incisions minimized risks such as wound infections and skin necrosis (8).

Limitations of the SP-technique might include extended operative times, especially during the learning phase, and the absence of long-term oncological data. Additionally, complex cases requiring concurrent pelvic lymphadenectomy may necessitate repositioning the robotic system, increasing procedure time.

**Conclusions:**

SP robotic-assisted ILND can represent a significant advancement in the surgical management of penile cancer, combining oncological safety with reduced surgical morbidity. Future studies are needed to validate these findings, compare surgical outcomes, and assess long-term efficacy.

Available at: http://www.intbrazjurol.com.br/video-section/20240663_Spena_et_al

## Data Availability

Data relating to this research can be found at: https://zenodo.org/records/14499695
